# Utility of Dental Operating Microscopes in Assessing Microleakage of Nanohybrid Resin Restorations Using Different Placement Techniques

**DOI:** 10.7759/cureus.28420

**Published:** 2022-08-26

**Authors:** Shweta Sedani, Anuja Ikhar, Pavan Bajaj, Pradnya Nikhade, Manoj Chandak

**Affiliations:** 1 Department of Conservative Dentistry and Endodontics, Sharad Pawar Dental College and Hospital, Datta Meghe Institute of Medical Sciences, Wardha, IND; 2 Department of Periodontics, Sharad Pawar Dental College and Hospital, Datta Meghe Institute of Medical Sciences, Wardha, IND

**Keywords:** composite placement techniques, oblique layering, split horizontal incremental, dental operating microscope, composite resin

## Abstract

Background

Composite resin has become a material of choice due to its aesthetic potential and durability. It is at a lower cost compared to ceramic restorations. But it has a tendency to shrink during polymerization, leading to the formation of gaps at the margins. Placement techniques of restoration can be modified to reduce stress shrinkage. A dental operating microscope will help in the precise and thin layering of composite against the cavity wall and the matrix band.

Methodology

Class I cavities were prepared in 30 human permanent premolars. All cavity surfaces were dried and acid-etched. A bonding agent was applied and they were light-cured. Specimens were divided into three groups: I, II, and III, where restorations were performed using horizontal layering, oblique layering, and split-increment horizontal layering techniques respectively. Samples were then submerged in silver nitrate solution and were examined under a stereomicroscope after a longitudinal section and graded for dye penetration.

Results

The dye penetration scores were subjected to a statistical analysis using Analysis of variance (ANOVA) with post hoc Tukey’s test with the statistical software Statistical Package for Social Sciences (SPSS version 12). The level of significance was set at 0.05 for all statistical inferences.

Conclusion

The split-increment horizontal layering technique under the dental operating microscope showed less microleakage when compared to other methods and is the preferred method for composite restorations.

## Introduction

Due to the increasing awareness and concern for aesthetic dentistry, composite resin has become the material of choice for anterior and posterior restorations. Although resins come with many advantages, they have a tendency to shrink during polymerisation by about 2% to 7%, leading to the generation of stress at the interface of the tooth and composite material; a major disadvantage leading to failure of adhesion and cohesion [1]. This may lead to the formation of gaps at the margins. These gaps when filled with oral fluids and bacteria, lead to microleakage with the potential for development of sensitivity, secondary caries, damage to the pulp, and discolouration of the margin.

The configuration factor, techniques of restoration, degree of conversion, the content of the filler, elastic modulus, light‑curing variables, water sorption, and the influence of substrate are the various factors which usually affect polymerisation shrinkage [2]. Amongst these factors, the placement techniques of restoration are one of the important factors and can be modified to reduce stress shrinkage. Further, refinement can be done by using magnification techniques in the form of a dental operating microscope, which will help reduce human errors and can help in the meticulous and thin layering of composite against the walls of the cavity [3].

Hence, this study aimed to assess the microleakage of nanohybrid resin restorations amongst three different composite placement techniques with the use of a dental operating microscope.

## Materials and methods

Thirty human permanent premolars were collected for the study, all of which were mandibular premolars with intact crowns that were devoid of cavities, cracks, or any restorations and were extracted for periodontal reasons. All the teeth were ultrasonically cleaned and preserved in normal saline. Using a diamond bur, Class I cavities on the occlusal surfaces of all the teeth were created according to GV Black principles (round bur - BR-41, straight bur - SF -31). All cavity surfaces were dried with compressed air before being acid-etched for 15 seconds with Prime Dental India's 37 per cent phosphoric acid. After that, all the cavities were rinsed with water for 15 seconds. Dry cotton pellets were used to dry the cavities once again. The bonding agent (3M ESPE) was applied to the floor and walls of all the cavities with an applicator tip and gently dried for five seconds. Following that, the cavities were light-cured for 20 seconds as directed by the manufacturer.

The specimens were divided into three groups where restorations were performed using horizontal layering, oblique layering, and split-increment horizontal layering techniques respectively. 

**Figure 1:**
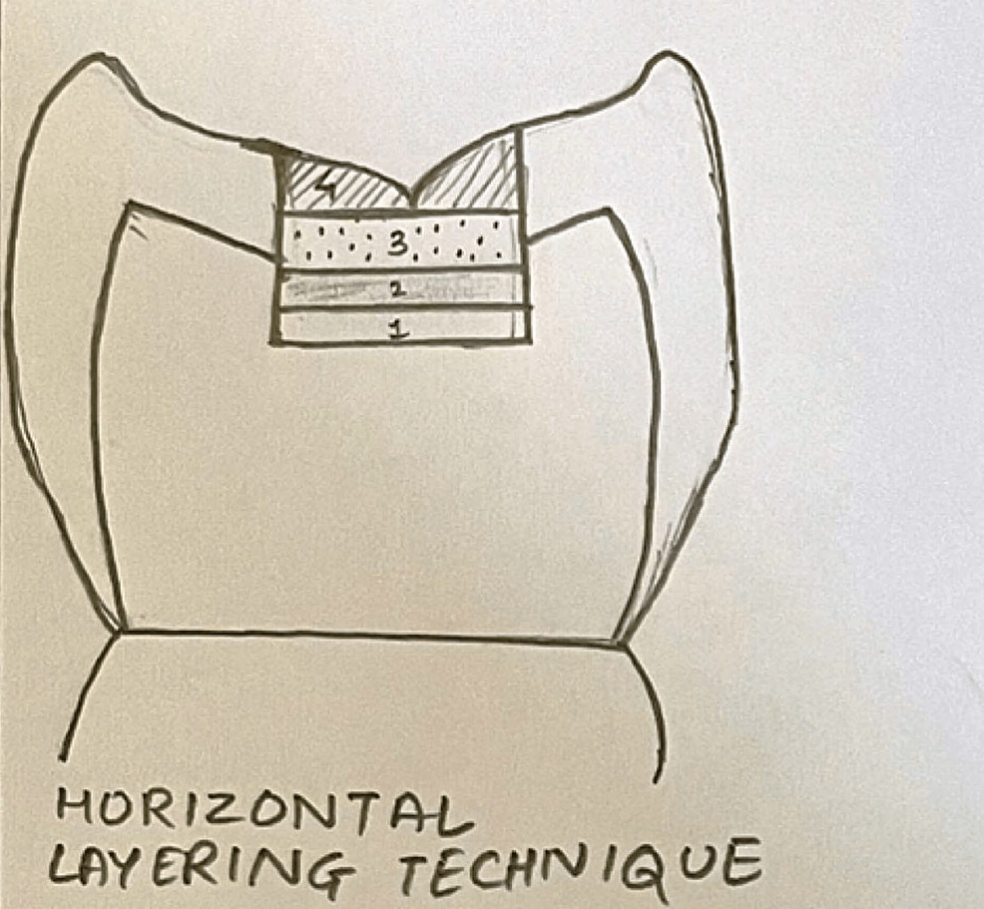
Composite placement technique in Class I cavity using the horizontal layering technique Figure created by author Shweta Sedani

In Group I, restoration was done by using the horizontal layering technique (n = 10) (Figure 1).

**Figure 2:**
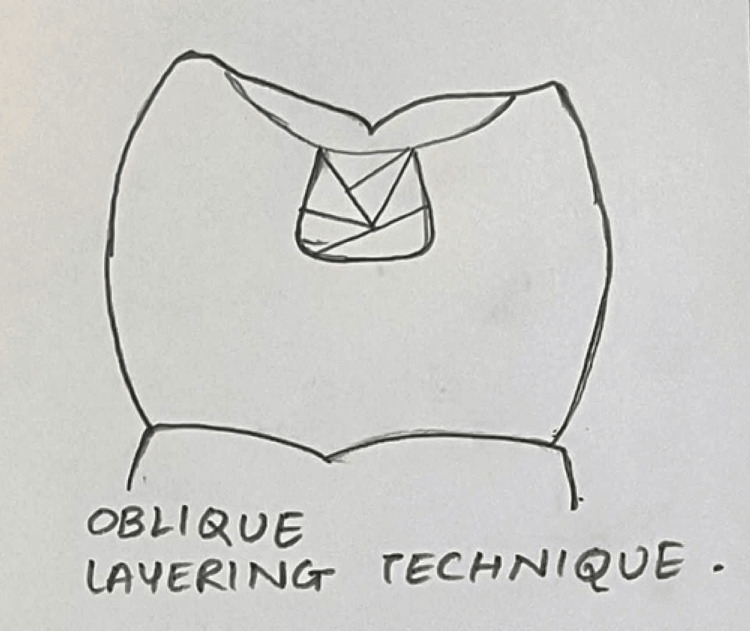
Composite placement technique in Class I cavity using the oblique layering technique Figure created by author Shweta Sedani

In Group II, restoration was done by using the oblique layering technique (n = 10) (Figure 2).

**Figure 3:**
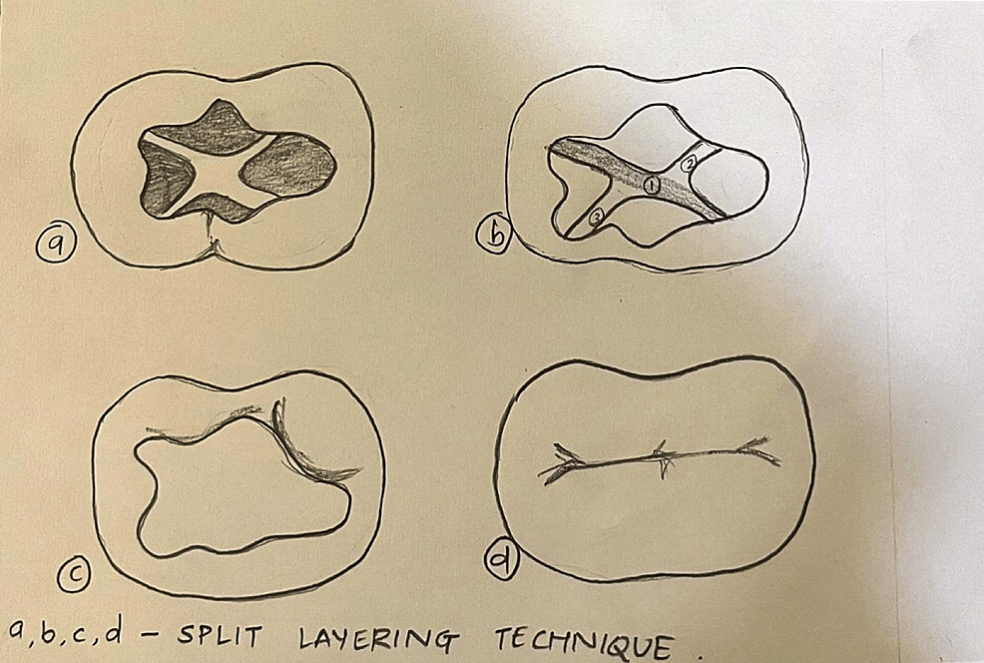
Composite placement technique in Class I cavity using the split-increment horizontal technique Figure created by author Shweta Sedani

In Group III, restoration was done by using the split-increment horizontal technique (n = 10) (Figure 3).

Group I: Horizontal Layering Technique

The horizontal layering placement approach makes use of 1 mm thick composite resin layers. According to the reports, this approach increases the configuration factor (C-factor), which raises shrinkage stress. The composite restorative material (3M™ Paradigm™ Nano Hybrid Universal Restorative) was filled up to half of the depth of the cavity which was light-cured for 40 seconds. The second increment filled the remainder of the cavity up to the cavosurface margin. It was further light-cured for 40 seconds.

Group II: Oblique Layering Technique

A succession of composite increments in the shape of a wedge is used in this technique. This approach lowers the C-factor and prevents cavity wall distortion. To drive the polymerisation vectors toward the adhesive surface, each increment underwent photocuring twice, initially through the cavity walls, followed by the occlusal surface.

Group III: Split-Increment Horizontal Technique

The composite was placed up to half of the depth of the cavity. This was followed by performing two diagonal slices with the help of a blunt probe up to the entire depth of the cavity. This led to the splitting of the uncured increment into four triangular-shaped portions. The light curing was then performed for 40 seconds. The second increment was then positioned into the diagonal cuts and was light-cured. This was followed by the third increment that was placed to fill the rest of the cavity up to the cavosurface edge, which was light-cured for another 40 seconds.

This procedure reduces the impact of polymerisation shrinkage on cavity walls and adhesive surfaces. The C-factor ratio is reduced from five, which is the greatest and least favourable, to 0.5, which is the second most favourable. Free composite surface flowing at cuts rather than at the bonded interfaces relieves shrinkage stress in such small increment parts with a low configuration factor ratio, decreasing the negative impact of polymerisation shrinkage stresses. Composite finishing burs were used to complete and polish all of the restorations. The above procedures, from the preparation of Class I cavities to the restoration by each incremental technique and polishing, were performed under a dental operating microscope. All the samples were stored in distilled water in separate containers at room temperature for 24 hours to allow for delayed polymerisation shrinkage of the composite restorations. The surfaces of the tooth specimens were covered with nail varnish after they were removed from normal saline and dried. The surfaces were saved for 2 mm around the restoration to allow contact with the tracing agent.

Teeth samples were then placed in different colour bags according to the experimental groups for identification. Porous bags were placed in a thermocycling machine. Samples were thermocycled for 1500 cycles between 12 and 60 degrees with a 30-second immersion and a 10-second interval between the baths. This procedure was performed to simulate the oral environment since there is a direct relationship between microleakage and thermal changes in composite restorations.

All the specimens were submerged for 24 hours in 50 per cent silver nitrate solution after thermocycling. Following this technique, all the specimens were carefully cleaned with distilled water. The nail varnish was removed using acetone. The samples were then air-dried after this treatment. The samples were then sectioned longitudinally in a buccolingual direction at a slow speed using a diamond disc with water cooling in a micromotor straight handpiece. After that, all three groups' specimens were examined under a stereomicroscope (x 30 magnification) and graded for dye penetration.

**Figure 4:**
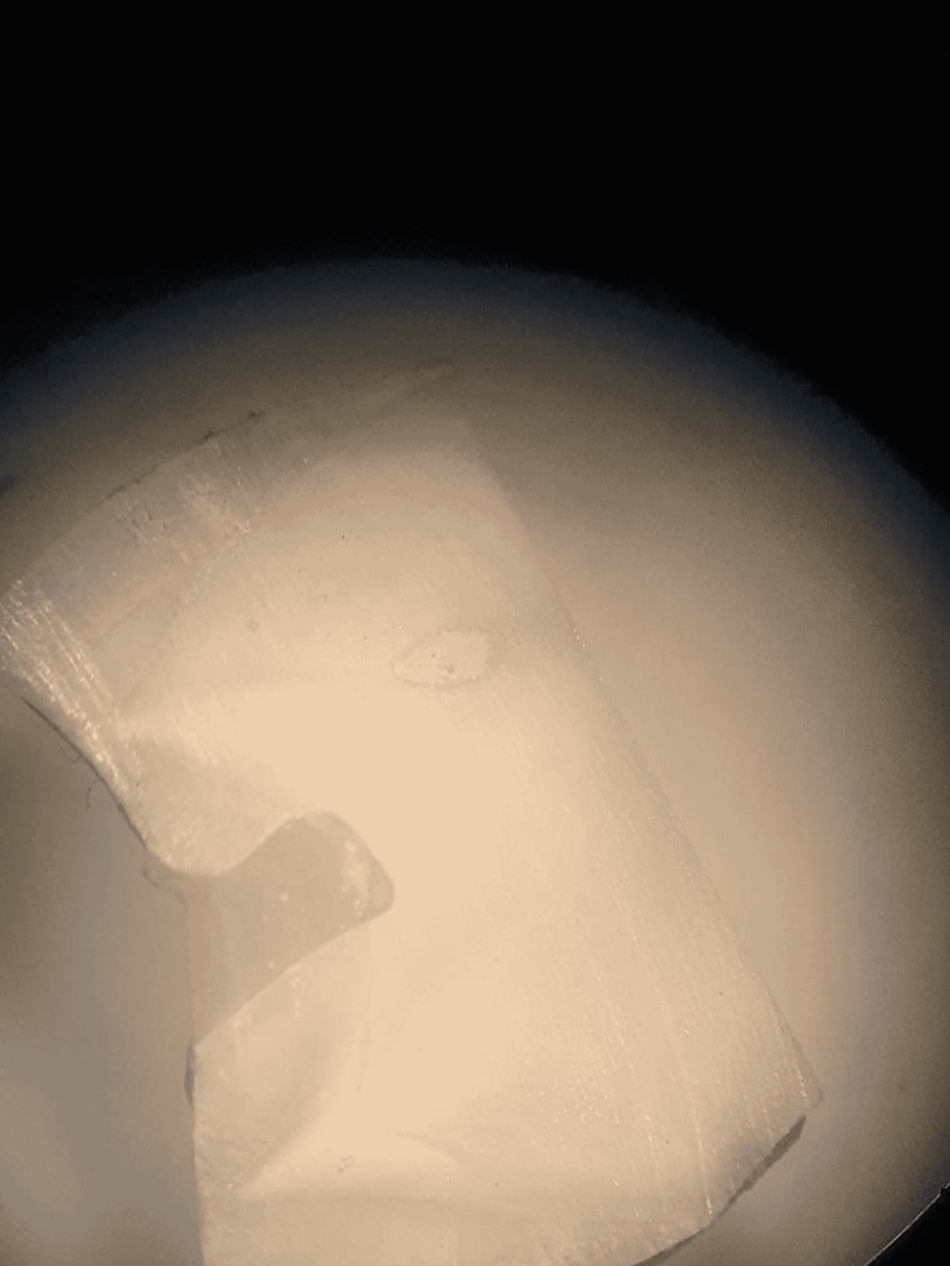
Specimen with dye penetration score as 0

**Figure 5:**
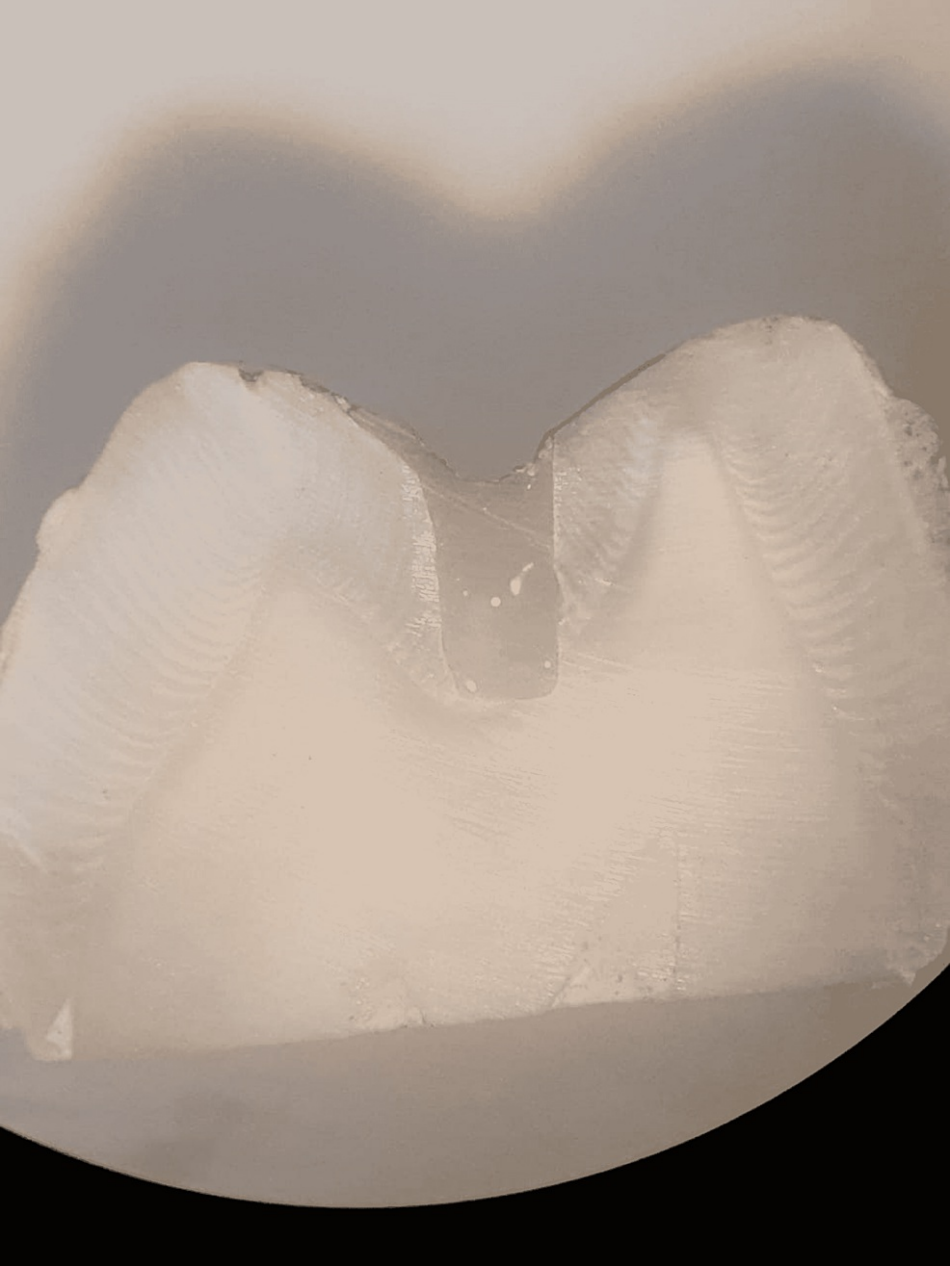
Specimen with dye penetration score as 1

The following scoring criteria for the assessment of microleakage were carried out:

0 = No penetration of dye; 1 = Dye penetration up to 1/3 of the cavity depth; 2 = Dye penetration up to 2/3 of the cavity depth; 3 = Dye penetration up to the full depth of the cavity; 4 = Dye penetration onto the pulpal floor (See Figures 4, 5).

The resultant microleakage scores were analysed by analysis of variance (ANOVA )and post hoc Tukey’s statistical tests.

## Results

The dye penetration scores were subjected to a statistical analysis using the statistical software Statistical Package for Social Sciences (SPSS version 12). The level of significance was set to 0.05 for all statistical inferences. ANOVA with post hoc Tukey’s statistical test was applied to test the difference between groups. Mean, standard deviation, minimum and maximum values of dye penetration scores for Group I, Group II, and Group III are depicted in Table 1.

**Table 1:** Mean, standard deviation, minimum and maximum values of dye penetration scores for Group I, Group II, and Group III

Group	Sample size	Mean	Standard deviation	Minimum	Maximum
I	10	0.8	0.78	0	2
II	10	0.6	0.69	0	2
III	10	0.2	0.42	0	1

The mean value of microleakage was 0.8 for Group I (horizontal layering technique), 0.6 for Group II (oblique layering technique) and it was 0.2 for Group III (split-increment horizontal technique) with the standard deviation of 0.78, 0,69, & 0.42 respectively. Table 2 depicts the p-value and F-value for all three groups as 0.133 & 2.17 respectively.

**Table 2:** P-value and F-value for Group I, Group II, and Group III

Parameters	Group I	Group II	Group III
Mean	0.8	0.6	0.2
Standard Deviation	0.78	0.69	0.42
F-Value	2.17		
p-Value	0.133		

The difference in the mean values of Group I (0.8 ± 0.78) and Group II (0.6 ± 0.69) was found to be 0.2 ± 1.03 which is non-significant with the p-value of 0.76 (Table 3).

**Table 3:** The comparison of the microleakage scores of Group I & Group II

Parameters	Group-I	Group-II	Difference	p-value
Microleakage	0.8 ± 0.78	0.6 ± 0.69	0.2 ± 1.03	0.76

The difference in the mean values of Group I (0.8 ± 0.78) and Group III (0.2 ± 0.42) was found to be 0.6 ± 0.96 which is non-significant with the p-value of 0.12 (Table 4).

**Table 4:** The comparison of the microleakage scores of Group I & Group III

Parameters	Group-I	Group-III	Difference	p-value
Microleakage	0.8 ± 0.78	0.2 ± 0.42	0.6 ± 0.96	0.12

The difference in the mean values of Group II (0.6 ± 0.69) and Group III (0.2 ± 0.42) was found to be 0.4 ± 0.84 which is non-significant with the p-value of 0.37 (Table 5).

**Table 5:** The comparison of the microleakage scores of Group II & Group III

Parameters	Group-II	Group-III	Difference	p-value
Microleakage	0.6 ± 0.69	0.2 ± 0.42	0.4 ± 0.84	0.37

## Discussion

Although the basic principles of restorative dentistry have remained unchanged for many years, dentists are applying aesthetic, biological, and mechanical criteria more stringently. With the use of a dental operating microscope and other magnification equipment, including loupes, dentists are performing more aesthetic and precise treatments with a significant reduction in chair time as well as an improved success rate [4].

A dental microscope allows fine and precise movements by providing three to 20 times magnification. This high level of magnification also allows for assessing fine edges, including their morphology and position, which helps in knowing the position of restoration and the removal of excess adhesive [5]. It ensures 0.1 mm accuracy and has an impact on tooth preparation as well as the operator's ergonomic comfort. Under the magnification, good integrity is ensured because packing a thin layer of composite material evenly against the cavity walls and the matrix band is easy. Packing oblique layers of the composite up to the cavity margins without any excess material provides a restoration with improved physical as well as mechanical properties. It minimises the finishing procedures too. Hence, a microscope was used in our study to utilise its advantageous properties.

Currently, nanohybrid resin composites are the most popular restorative material because they have a combination of nanoparticles along with submicron particles that impart superior and improved chemical, mechanical, and optical properties. They increase the distribution of fillers in the matrix but also come with the major concern of stress and polymerisation shrinkage. Monomer molecules of dental resin composites undergo polymerisation shrinkage while forming a polymer network, which results in contraction stresses causing microleakage and internal stress in the tooth structure [6-10].

Microleakage is the diffusion of bacteria and oral fluids between the cavity walls and the restorative material. Marginal discolouration, postoperative sensitivity, secondary caries, and pulp damage are all symptoms [11].

Efforts have been made to reduce polymerisation shrinkage by reducing the configuration factor, reducing the filler content of the composite material, and switching to various layering placement techniques.

In this study, we aimed at evaluating various layering placement techniques to reduce microleakage using a dental operating microscope.

In our study, the mean values of the microleakage for the horizontal layering group, the oblique layering group, and the split-increment horizontal group were 0.8, 0.6, and 0.2 respectively. The horizontal layering technique had the highest microleakage which was followed by the oblique layering technique. The least microleakage was found with the split- increment horizontal technique under the dental operating microscope, though not significant statistically.

The horizontal layering technique showed higher microleakage scores compared to other techniques when performed under a microscope, though not significant statistically. Gupta M et al. assessed leakage in primary molars with respect to different placement techniques in teeth restored with compomer and found that leakage exhibited in horizontal and oblique layering techniques was 56.6% and 46.6% respectively, which was not significant statistically [12]. Similar results were obtained in our study when we compared the two techniques (Table 3).

Polymerisation shrinkage takes place in each increment in the horizontal layering technique as the composite is applied incrementally. A thin layer of composite creates less tensile force, lowers the C factor, and reduces the stress caused by polymerisation shrinkage.

The contraction forces generated between the opposite walls can be damaging to the restoration by building up stress, gapping, and formation of cuspal fissures which can be limited by using the oblique layering technique. This technique helps to reduce the C-factor and prevents cavity distortion [13-15].

When Group I and Group III were compared, the difference in the mean values of Group I (0.8 ± 0.78) and Group III (0.2 ± 0.42) was found to be 0.6 ± 0.96, which is non-significant with a p-value of 0.12. The microleakage score was found to be higher with the horizontal layering technique than with the split-increment horizontal technique when performed under a dental operating microscope, though not significant statistically. The results of our study are in agreement with the results of Somani et al., where it was found that although microleakage was present in all the techniques used by them, the split-increment horizontal technique was found to be better, though not significant statistically [1].

New unbonded composite surfaces are created by cutting the flat composite increment diagonally into two triangular composite sections to act as a reservoir for plastic deformation during light polymerisation in the split-increment horizontal technique. As a result, the restoration's interfacial bond and marginal integrity are preserved. Prior to photocuring, the large horizontal increment in the proximal and occlusal portions of cavities can be split into smaller triangular parts to reduce polymerization shrinkage.

Al-Zahawi et al. reported similar findings in accordance with the results of the current study. Their findings showed that gingival microleakage is less with nanohybrid composites than with microhybrid composites and that the bulk technique is the worst in both groups, followed by the vertical, divided horizontal, and/or centripetal approaches [16]. According to Al-Zahawi et al., the split-increment portions of the composite's contact with two opposing cavity walls will be prevented when the diagonal cuts are made in composite increment [16]. This will reduce the free flow of the composite surface at the diagonal cuts and also decrease the negative effects of polymerization shrinkage stresses on the walls of the cavity as well as the adhesive interface. 

When Group II and Group III were compared, the difference in the mean values of Group II (0.6 ± 0.69) and Group III (0.2 ± 0.42) was found to be 0.4 ± 0.84, which is non-significant with a p-value of 0.37. Microleakage scores were found to be higher with the oblique layering technique than with the split-increment horizontal technique when performed under a dental operating microscope, though not significant statistically. The findings of our study are similar to the findings of Nadig R et al, who investigated the influence of several placement procedures (bulk, centripetal, oblique layering, and split-increment horizontal) on microleakage in Class II resin restorations [17]. Scores of microleakage showed that the incremental technique was superior to bulk and the split-increment horizontal approach was the best among incremental techniques. The split-increment horizontal technique was shown to have the lowest microleakage rates among incremental procedures.

Hence, from this study, it can be inferred that all the placement techniques have exhibited some amount of microleakage, though the technique of placement had no statistically significant difference. It was also found that the overall score of microleakage was lower in all the techniques confirming the utility of a dental operating microscope as a tool which achieves precision with its meticulous use. A dental operating microscope can be used routinely in the practice to achieve success in composite restorations.

Hence, the split-increment horizontal placement technique performed under a dental operating microscope can be used to restore Class I cavities.

Limitations of this study include that the comparison was not made with the conventional method (direct vision). Therefore, it is questionable whether the study results are attributable to the placement technique or to the use of a microscope. Also, the results of this study were specific to the material that was used. Another limitation is that the study only revealed the significance of the three layering techniques that were used, and since it was in vitro, it did not mimic clinical conditions.

## Conclusions

Neither of the placement techniques tested showed a complete absence of leakage. However, the split-increment horizontal placement technique under the dental operating microscope is the preferred method for composite restorations. It can also be mentioned that therapy provided with a dental operating microscope provides higher care for patients and is more effective.
